# Implications of serum uric acid for female infertility: results from the national health and nutrition examination survey, 2013–2020

**DOI:** 10.1186/s12905-023-02234-1

**Published:** 2023-03-11

**Authors:** Jiemei Liang, Xiting Chen, Jinfa Huang, Weizhe Nie, Qian Yang, Qitao Huang, Kaixian Deng

**Affiliations:** Department of Gynaecology, Shunde Hospital of Southern Medical University (The First People’s Hospital of Shunde Foshan), No. 1, Jiazi Road, Lunjiao Town, Shunde District, Foshan, 528300 Guangdong China

**Keywords:** Serum uric acid, Infertility, Female fertility status, NHANES

## Abstract

**Background:**

There is limited concrete evidence connecting serum uric acid levels to female infertility. Therefore, this study aimed to find out if serum uric acid levels are independently related to female infertility.

**Methods:**

From the National Health and Nutrition Examination Survey (NHANES) 2013–2020, a total sample of 5872 chosen female participants between the ages of 18 and 49 were identified for this cross-sectional study. The serum uric acid levels (mg/dL) of each participant were tested, and the reproductive health questionnaire was used to evaluate each subject's reproductive status. Both in the analyses of the full sample and each subgroup, logistic regression models were used to evaluate the relationship between the two variables. A stratified multivariate logistic regression model was used to perform the subgroup analysis based on serum uric acid levels.

**Results:**

Infertility was found in 649 (11.1%) of the 5,872 female adults in this study, with greater mean serum uric acid levels (4.7 mg/dL vs. 4.5 mg/dL). Serum uric acid levels were associated with infertility in both the initial and adjusted models. According to multivariate logistic regression, the odds of female infertility were found to be significantly higher with rising serum uric acid levels (Q4 [≥ 5.2 mg/dL] vs. Q1 [≤ 3.6 mg/dL]), adjusted odds ratio [aOR] = 1.59, *p* = 0.002]. The data suggests that there is a dose–response relationship between the two.

**Conclusions:**

The results from this nationally representative sample from the United States confirmed the idea that there is a link between increased serum uric acid levels and female infertility. Future research is necessary to evaluate the relationship between serum uric acid levels and female infertility and explicate the underlying mechanisms of this relationship.

## Background

Infertility is defined as the inability to conceive after unprotected sexual activity or therapeutic donor insemination in women under the age of 35 or within six months in women over the age of 35. Infertility is estimated to affect 15% of all couples worldwide [1, 2]. The World Health Organization has classified infertility as a social disease, and the Centres for Disease Control and Prevention (CDC) in the United States have named infertility a public health priority [3]. Infertility is more than just a quality-of-life concern and has significant public health repercussions, such as psychological discomfort, social stigmatisation, economic pressure, and marital discord [4, 5].

Serum uric acid (SUA) is a major by-product of purine metabolism catalysed by xanthine oxidoreductase (XOR). XOR is a source of reactive oxygen species, which can lead to oxidative stress and endothelial dysfunction. When SUA becomes an oxidant, it contributes to the development of various pathological processes in the body that are ruled by oxidative stress [6]. SUA might behave as an antioxidant, which might have some protective effects, or as a pro-oxidant, which might accelerate a chain reaction of free radicals and cause oxidative damage to cells [7]. In addition to inducing oxidative stress, SUA has a role in the metabolism of lipids, glucose, and inflammation [8, 9]. Hyperuricemia (HUA) is a chronic metabolic condition, defined by unusually elevated SUA levels, which has been identified to have effects on multiple body organs through its numerous effects and contribute to the emergence of several disease states [10]. In the female reproductive system, hyperuricemia with SUA deposition may cause female sexual dysfunction [11]. According to research on buffalo ovaries, disruption of the plasma-follicular barrier structure is linked to higher levels of SUA [12]. Oocyte meiosis can be inhibited by hypoxanthine, which is a precursor to SUA [13]. SUA has potential mechanisms such as oxidative stress, promotion of inflammation, endothelial damage and thrombosis, and therefore, high levels of SUA may be correlated with incread clinical severity of polycystic ovarian syndrome (PCOS), endometriosis, or adverse pregnancy outcomes [14–17].

Our hominoid ancestors had gene alterations that led to the lack of uricase. As a consequence, humans must adapt to SUA levels that are comparatively greater [18]. In addition to genes, the risk of hyperuricemia is associated with ethnicity, age, lifestyle, and dietary factors [19]. Unhealthy living and eating habits in modern society lead to an increased incidence of hyperuricemia. High SUA levels are involved in the development of several diseases, including obesity, diabetes, metabolic syndrome, kidney disease, cardiovascular disease, and female reproductive disorders [10, 17, 20–22]. At the same time, unhealthy lifestyle and dietary factors will increase the prevalence of female infertility [23, 24]. Hence, we hypothesise that increased SUA levels may lead to decreased female fertility. Ultimately, this leads to an increased incidence of female infertility.

There are no studies that we are aware of that used a nationally representative sample to study the link between SUA levels and female infertility. In this cross-sectional study, we identified and examined correlations between SUA levels and female infertility using the latest nationally representative data from the National Health and Nutrition Examination Survey (NHANES) 2013–2020.

## Methods

### Data sources and study population

The National Centre for Health Statistics (NCHS) at the Centers for Disease Control and Prevention (CDC) collects data on nutritional status and health information for the NHANES which is a national population-based survey. All data for this study were provided in NHANES cycles 2013–2020. We used this data to describe the demographic characteristics of our population, obtain female self-reported infertility rates, and assess SUA levels in women 18 to 49 years old. This study was based on NHANES public data, and all information was collected from the official website [25]. The NHANES protocols are approved by the NCHS Research Ethics Review Board, and every respondent provided their signed informed consent [26].

The study enrolled women aged 18–49 years old who completed an interview using the reproductive health questionnaire and had a physical examination at the mobile examination centre (MEC). A multistage, stratified probability strategy was used to choose survey respondents [27]. Demographic and health history information was obtained through an extensive household interview. Physical assessments included the collection of blood samples at the MEC. Samples of serum were examined by the CDC Division of Laboratory Sciences. Analyses of the samples were performed in the United States.

### Fertility assessment

Responses from the reproductive health questionnaire were used to calculate the dependent variable of infertility (variable name in the questionnaire: RHQ074). Those who answered affirmatively to the survey question, "Have you ever attempted to become pregnant for at least a year without becoming pregnant?" were presumed to have infertility [28].

### Measurement and classification of SUA

The main independent variable was SUA measured in mg/dL. SUA levels were collected during subject enrolment in the NHANES using a colorimetric method in which uricase oxidises UA to allantoin and hydrogen peroxide (the Beckman Coulter UniCel DxC 800 Synchron chemistry analyzer between 2013 to 2014, the Beckman Coulter UniCel DxC 800 Synchron and the Beckman Coulter UniCel DxC 660i Synchron since 2015). Quality-control procedures' specifics have been disclosed elsewhere [29]. Values are reported in mg/dL and can be converted to μmol/L by multiplying by 59.48.

### Covariates

In the NHANES database, factors were classified as demographics or possible confounders that could influence SUA or fertility status [30, 31]. We considered demographic characteristics (age, sex, race or ethnicity, education, marital status, and ratio of family income to poverty); lifestyles (drinking and smoking); health insurance coverage; physical examinations; and laboratory tests (serum lipids, creatinine (Cr), blood urea nitrogen(BUN), and estimated glomerular filtration rate (eGFR)). In addition, we considered body mass index (BMI) and waist circumference (WC). Disease history was also taken into account and included the following diagnoses: hypertension [32] (characterised as being on anti-hypertensive medication and having a systolic blood pressure ≤ 140 mmHg or a diastolic blood pressure ≤ 90 mmHg); diabetes mellitus [32] (obtained through self-report and using diabetes medications); and metabolic syndrome (MetS). BMI was coded into three categories [33]: (underweight or normal weight (< 25 kg/m^2^), overweight (25–29.9 kg/m^2^), and obese (> 30 kg/m^2^). MetS was diagnosed in respondents when at least three of the following five symptoms were present [34]: WC ≥ 88 cm, triglycerides (TG) ≥ 150 mg/dL, high-density lipoprotein (HDL) < 50 mg/dL, systolic blood pressure (SBP) ≥ 130 mmHg or diastolic blood pressure (DBP) ≥ 85 mmHg (averaged over three readings), or fasting plasma glucose (FPG) ≥ 100 mg/dL. CKD-EPI Creatinine Eq. (2021): [35].

$$eGFR = {142} \times {\text{min}}\left( {{\text{standardised}}\frac{{{\text{S}}_{{{\text{cr}}}} }}{{\text{k}}},{ }1} \right)^{ \propto } \times \max \left( {{\text{standardised}}\frac{{{\text{S}}_{{{\text{cr}}}} }}{{\text{k}}},{ }1} \right)^{ - 1.200} \times 0.9938 ^{Age} \times 1,012\left( {\text{if female}} \right)$$

### Statistical analysis

Means and standard errors (SE) were used for continuous variables, as well as percentages and standard errors for categorical variables. The t-test (normal distribution) and Kruskal–Wallis test (skewed distribution) were used to assess continuous variables. The stratified multivariate logistic regression model was used to perform the subgroup analysis by SUA levels. The relationship between SUA levels and infertility was investigated by using SUA data as a continuous variable and in quartiles. The odds ratios (ORs) and their 95% confidence intervals (CIs) were estimated. The following stratified multivariate logistic regression models were used to assess the effect of SUA levels on female infertility: Model 1: no adjustment; Model 2: adjusted for social demographic covariables (age, race/ethnicity, education, PIR) and health insurance coverage; Model 3: adjusted for the variables in Model 2 plus BUN, Cr, and eGFR; Model 4: adjusted for the variables in Model 3 plus BMI, DM, hypertension, and MetS.

All statistical analyses were carried out using the software tools R, version 4.1.1 (http://www.R-project.org, The R Foundation), and Free Statistics, version 1.5. In all tests, a statistically significant difference was defined as *P* < 0.05 (two-sided).

## Results

**Fig. 1:**
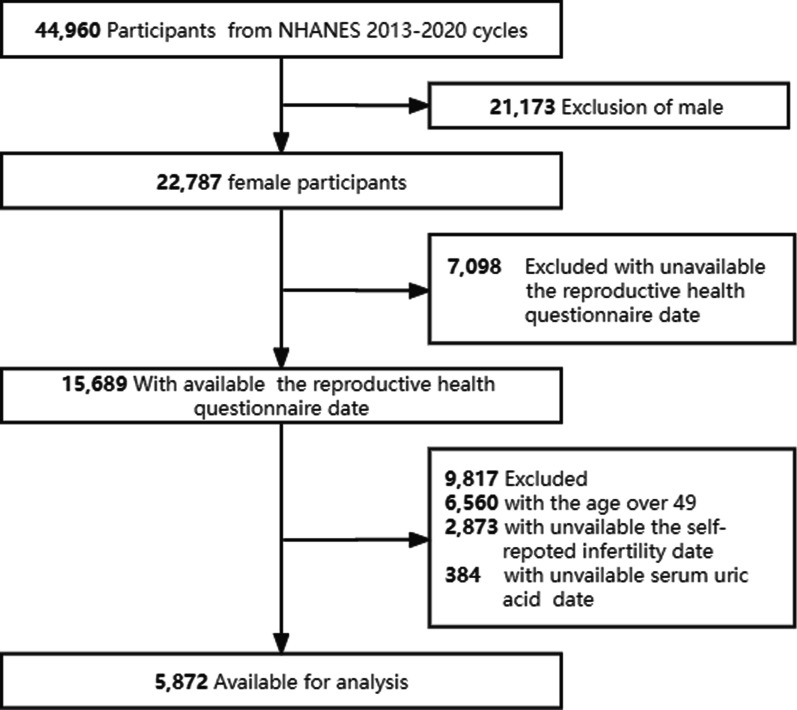
Flow chart of sample selection

Four cycles of NHANES (2013–2014, 2015–2016, 2017–2018, and 2017–2020) were used in this study. There were 44,960 eligible participants, and of these, 22,673 adult females completed the interview and 15,689 participants completed the reproductive health questionnaire. Participants with missing data in SUA or answering the fertility information for RHQ074 variables (*n* = 9,817) were excluded. Our analyses included the remaining 5,872 participants aged 18–49. Figure 1 shows the flowchart of the exclusion criteria.

Table 1 presents the descriptive characteristics of the study population according to their fertility status. Infertility was projected to affect 11.1% of women between the ages of 18 and 49. Infertile women were older (36.2 years vs. 32.9 years), their SUA mean was more significant (4.7 mg/dL vs. 4.5 mg/dL), they had higher PIR levels (2.6 vs. 2.3), and they had lower eGFR levels (108.5 mL/min/1.73 m2 vs. 111.0 mL/min/1.73 m2) than women with non-infertility. Female participants with infertility were more likely to have a regular partner (14.8% vs. 8.1%), have been pregnant at least once (13.3% vs. 7.4%), have obesity (14.3% vs. 8.6%), have hyperuricemia (13.8% vs. 10.7%), diabetes mellitus (8.8% vs. 4.6%), MetS (12.3% vs. 9.2%), and have hypertension (21.5% vs. 15.5%). There were no statistical differences in ethnicity, education, health insurance coverage, BUN, or Cr.

**Table 1:** Baseline characteristics of participants

Covariates	Total (*n* = 5,872)	Infertile (*n* = 649)	Fertile (*n* = 5,223)	*P*-value
*Age, years*	33.3 ± 9.4	36.2 ± 8.0	32.9 ± 9.5	< 0.001
Age < 35	3,127 (53.3)	259 (39.9)	2,868 (54.9)	
Age ≥ 35	2,745 (46.7)	390 (60.1)	2,355 (45.1)	
*Race/ethnicity*				0.058
Mexican American	972 (16.6)	88 (13.6)	884 (16.9)	
Other Hispanic	602 (10.3)	62 (9.6)	540 (10.3)	
Non-Hispanic white	1,922 (32.7)	246 (37.9)	1,676 (32.1)	
Non-Hispanic black	1,350 (23.0)	143 (22)	1,207 (23.1)	
Non-Hispanic asian	718 (12.2)	77 (11.9)	641 (12.3)	
Other race	308 (5.2)	33 (5.1)	275 (5.3)	
*Education*				0.695
Less than high school	1,875 (35.1)	215 (33.8)	1,660 (35.3)	
High school	1,989 (37.3)	239 (37.5)	1,750 (37.2)	
More than high school	1,474 (27.6)	183 (28.7)	1,291 (27.5)	
*Marital status*				< 0.001
Live alone	1,465 (41.3)	118 (27.6)	1,347 (43.2)	
Married or cohabiting	2,082 (58.7)	309 (72.4)	1,773 (56.8)	
PIR	2.3 ± 1.6	2.6 ± 1.6	2.3 ± 1.6	< 0.001
*Health insurance coverage*				0.989
Yes	4,639 (79.1)	512 (79)	4,127 (79.1)	
No	1,225 (20.9)	136 (21)	1,089 (20.9)	
*Drinking*				0.003
Yes	1,700 (62.4)	224 (70)	1,476 (61.3)	
No	1,026 (37.6)	96 (30)	930 (38.7)	
*Smoking*				< 0.001
Yes	1,672 (28.5)	238 (36.7)	1,434 (27.5)	
No	4,198 (71.5)	411 (63.3)	3,787 (72.5)	
SUA, mg/dL	4.5 ± 1.1	4.7 ± 1.1	4.5 ± 1.1	< 0.001
*Hyperuricemia*				0.026
Yes	600 (10.2)	83 (12.8)	517 (9.9)	
No	5,272 (89.8)	566 (87.2)	4,706 (90.1)	
BUN, mg/dL	11.0 (9.0, 13.0)	11.0 (9.0, 14.0)	11.0 (9.0, 13.0)	0.719
Cr, mg/dL	0.7 (0.6, 0.8)	0.7 (0.6, 0.8)	0.7 (0.6, 0.8)	0.473
eGFR, mL/min/1.73 m^2^	110.7 ± 16.7	108.5 ± 16.2	111.0 ± 16.8	< 0.001
BMI, kg/m^2^	29.8 ± 8.5	32.0 ± 9.0	29.5 ± 8.4	< 0.001
*Obesity*				< 0.001
Yes	2,465 (42.4)	352 (55)	2,113 (40.8)	
No	3,352 (57.6)	288 (45)	3,064 (59.2)	
*Diabetes Mellitus*				< 0.001
Yes	295 (5.0)	57 (8.8)	238 (4.6)	
No	5,573 (95.0)	592 (91.2)	4,981 (95.4)	
*Hypertension*				< 0.001
Yes	950 (16.2)	140 (21.6)	810 (15.5)	
No	4,918 (83.8)	509 (78.4)	4,409 (84.5)	
*MetS*				0.012
Yes	559 (9.5)	80 (12.3)	479 (9.2)	
No	5,313 (90.5)	569 (87.7)	4,744 (90.8)	
*Ever been pregnant*				< 0.001
Ever	4,063 (76.1)	542 (85.2)	3,521 (74.9)	
Never	1,276 (23.9)	94 (14.8)	1,182 (25.1)	

Table 2 shows the outcomes of unweighted multivariable logistic regression studies examining the association between SUA levels and the likelihood of infertility. In the initial model, SUA levels were positively associated with female infertility (OR = 1.19; 95% CI: 1.11–1.288). In adjusted models, the connection between SUA levels and the risk of female infertility in women was still positive (Model 2: OR = 1.19, 95% CI: 1.1–1.28; Model 3: OR = 1.22, 95% Cl 1.13–1.32; Model 4: OR = 1.16, 95% Cl 1.06–1.27). Female infertility was 76% more likely for women with SUA in the highest quartile [OR = 1.76, *P* < 0.001], compared to 59% more likely in Model 4 [aOR = 1.59, *P* = 0.002].

**Table 2:** Relationship between SUA (mg/dL) and fertility or infertility

Exposure	Odds ratio (95% confidence interval)			
	Model 1 (*n* = 5,872)	Model 2 (*n* = 5,336)	Model 3 (*n* = 5,335)	Model 4 (*n* = 5,328)
SUA (mg/dL)	1.19 (1.11–1.28)	1.19 (1.1–1.28)	1.22 (1.13–1.32)	1.16 (1.06–1.27)
	< 0.001	< 0.001	< 0.001	0.001
Q1 (≤ 3.6)	1(Ref)	1(Ref)	1(Ref)	1(Ref)
Q2 (3.7–4.3)	1.14 (0.88–1.48)	1.15 (0.87–1.53)	1.15 (0.87–1.53)	1.12 (0.84–1.49)
	0.324	0.317	0.318	0.454
Q3 (4.4–5.1)	1.42 (1.11–1.82)	1.49 (1.14–1.94)	1.52 (1.16–1.98)	1.38 (1.04–1.82)
	0.005	0.003	0.002	0.024
Q4 (≥ 5.2)	1.76 (1.38–2.24)	1.8 (1.39–2.35)	1.86 (1.42–2.44)	1.59 (1.19–2.13)

Model 1: adjusted for none

Model 2: adjusted for social demographic covariables (age, race/ethnicity, education, and PIR) and health insurance coverage

Model 3: adjusted for: Model 2 + BUN + Cr + eGFR

Model 4: adjusted for: Model 3 + BMI + DM + hypertension + MetS

**Fig. 2:**
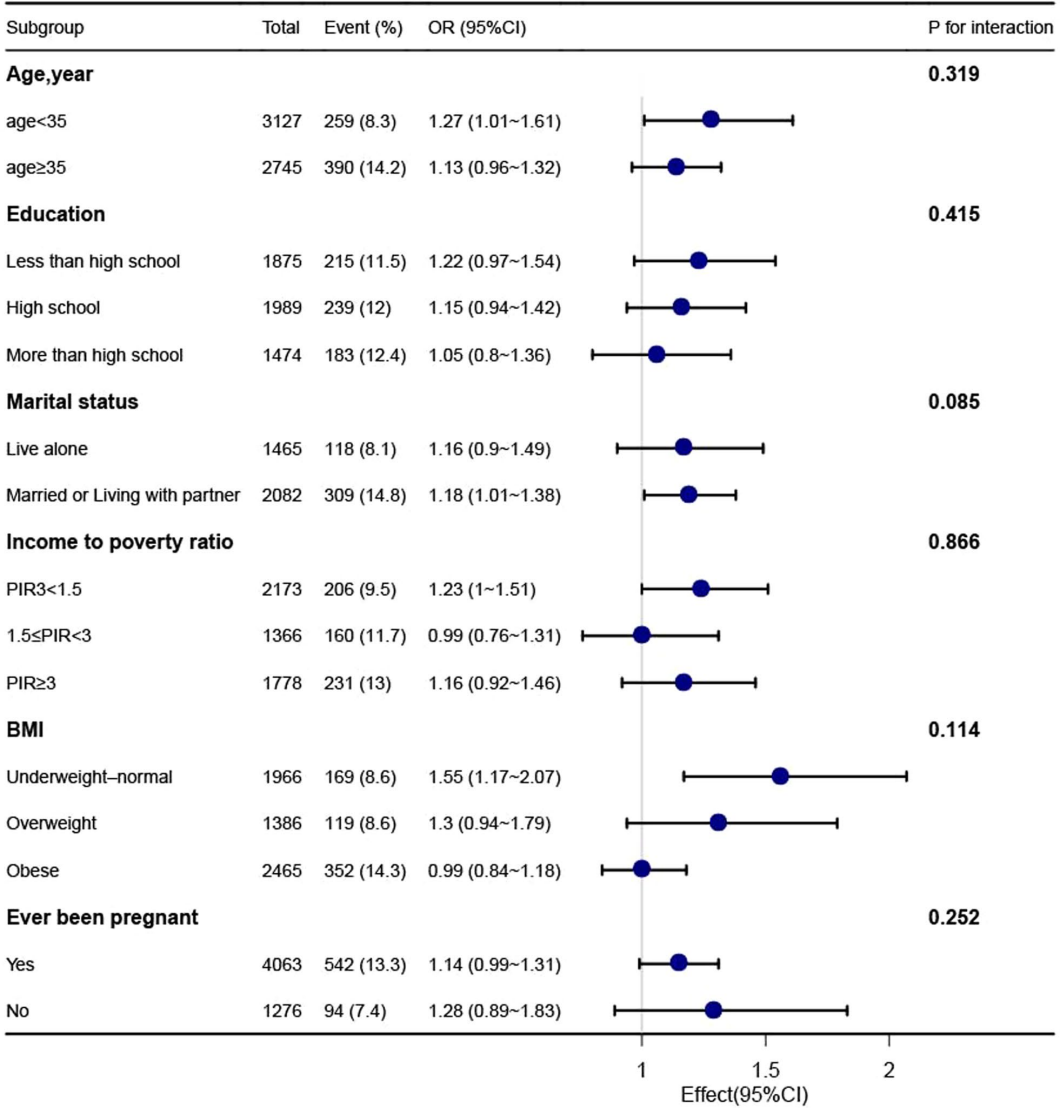
Association between SUA and infertility. Each stratification was adjusted for age, sex, race and ethnicity, educational level, marital status, family income, health insurance, drinking, smoking, BUN, Cr, eGFR, BMI, DM, hypertension, and MetS except for the stratification factor itself

In Fig. 2, the outcomes of the subgroup analysis are displayed. Participants aged 18 to 35 (OR = 1.27, 95% CI: 1.01–1.61), married or cohabiting (OR = 1.18, 95% CI: 1.01–1.38), below the poverty line (OR = 1.23, 95% CI: 1–1.51), and those without obesity (OR = 1.37, 95% CI: 1.12–1.68) showed the connection between infertility and SUA levels. Participants between the ages of 35 and 49, those who were single, lived above the poverty line, had never given birth, or belonged to the obesity categories, did not show any correlation.

## Discussion

In this nationally representative cross-sectional study, infertility was prevalent among women between the ages of 18 and 49 at an estimated 11.1%, which was within the range of the reported national prevalence (6.7%–15.5%) [36, 37]. There is a positive correlation between SUA and infertility among US female adults. In sensitivity studies, the magnitude and direction of this connection remained constant. The strength of the dose-dependent relationship between the SUA quartiles and infertility increased. The largest connection between infertility and SUA was seen among participants in the highest quartile (Q4). One of the interesting findings in this research is that the strength of the connection between SUA values and infertility grew in a dose-dependent manner. This was more notable in secondary infertility than primary infertility.

To the best of our knowledge, this is the first investigation into the relationship between SUA and infertility among American women of reproductive age who took part in the NHANES between 2013 and 2020. Studies on the connection between SUA and female infertility status are sparse and inconsistent. High SUA levels are associated with MetS, diabetes mellitus, cardiovascular disease, kidney disease, and female reproductive disorders [21, 22, 31, 38].

Previous studies have linked decreased fertility to increasing age, obesity, diabetes, MetS, hypertension, and gout [39–42]. Because SUA levels and female fertility are both associated with a variety of disease states, we were curious to see if there was a link between the two. Subfertility and infertility are terms that can be used interchangeably, and infertility is a disease that causes disability by impairing function [43]. Numerous plausible processes may underlie the link between SUA and infertility, according to earlier investigations. Previous studies have demonstrated that the antioxidant effect of physiological levels of SUA serves as an in vivo protective function [7]. When antioxidants like ascorbic acid are scarce, SUA can become an oxidant and participate in a variety of pathological processes caused by oxidative stress [6]. An imbalance between pro-oxidants and antioxidants can contribute to female reproductive difficulties like endometriosis, PCOS, and unexplained infertility [44]. In the reproductive system, excessive SUA levels are also linked to PCOS, endometriosis, pregnancy difficulties, adverse fatal outcomes, and other diseases [14, 16, 17, 22, 45]. As a result, we believe there is a link between SUA levels and female infertility.

Multiple body organs and systems can become damaged by an excessive buildup of SUA [38]. There is substantial evidence that uric acid levels can directly cause inflammation and abnormal lipid metabolism [8, 9]. Inflammatory pathways and hormonal aberrations are shared by reproductive illnesses (adenomyosis, endometriosis, uterine fibroids, and PCOS) and unexplained infertility and may reduce pregnancy success through shared processes [46]. Such processes could also contribute to the decreased fecundity of patients with endometriosis or POCS [47, 48]. Some infertility reasons have been connected to ovarian inflammation. According to one study, SUA levels have an impact on the quality of semen and male infertility [49]. It seems biologically plausible to have excessive SUA levels as a risk factor for excessive SUA levels and more studies are needed to confirm our findings and investigate the underlying mechanisms.

It has been proposed that dietary treatments provide a secure, economical means of controlling hyperuricemia. The Dietary Approaches to Stop Hypertension (DASH) diet and the Mediterranean diet, are well-known to help obtain optimal SUA levels, have differing SUA-lowering effects, and have been mentioned in previous reports on dietary styles restricting the consumption of fats and meats [50, 51]. Insulin resistance impairs glycolysis and the kidneys' ability to remove SUA, causing the increased synthesis of SUA and decreased urine SUA clearance [52]. According to a review, women had improved fertility when they followed healthy diets that prioritised fish, poultry, whole grains, fruits, and vegetables [24]. Excluding drug control, therapeutic lifestyle changes, appropriate weight loss, and adequate physical activity are beneficial to overall health while also improving hyperuricemia and infertility [53, 54]. For women who would like to become pregnant, paying attention to their nutrition and lifestyle choices, as well as their SUA levels, will help improve fertility.

This study had several strengths and limitations. The use of NHANES data is advantageous because it offers a nationally representative data set, extensive and detailed information on nutritional, demographic, and lifestyle factors, objective cognitive performance tests, and biological samples to control for known major confounders. The current study contains several drawbacks. First, because this research was cross-sectional, we were unable to draw any conclusions about the cause of the link between SUA and infertility in women of reproductive age. Second, women might not be able to recall exactly how long they attempted to get pregnant because infertility was measured through self-reporting. Thirdly, we could have missed infertile women who haven't attempted to become pregnant yet. Lastly, the study did not include data on concomitant gynaecological diseases (such as endometriosis, PCOS, fibroids, polyps, etc.), and we did not treat serum lipids as a covariate [55]. However, we did explore the potential influence of obesity and MetS on the association between SUA and infertility.

## Conclusions

According to the results of this cross-sectional investigation, SUA is positively correlated with infertility in the US adult female population. This discovery assists clinicians in consciously controlling SUA levels to decrease the rate of infertility.

## Data Availability

The datasets generated or analysed during the current study are included in this published article. Any further inquiries can be directed to the corresponding author.

## Abbreviations

AOR: Adjusted odds ratio.
BMI: Body mass index
BUN: Blood urea nitrogen
CDC: The centrescenters for disease control and prevention
CI: Confidence intervals
Cr: Creatinine
DBP: Diastolic blood pressure
EGFR: Estimated glomerular filtration rate
FPG: Fasting plasma glucose
HDL: High-density lipoprotein
HUA: Hyperuricemia
MEC: The mobile examination centrecenter
MetS: Metabolic syndrome
NCHS: National centrecenter for health statistics
NHANES: National health and nutrition examination survey
OR: Odds ratio
PCOS: Polycystic ovary syndrome
PIR: Ratio of family income to poverty
SBP: Systolic blood pressure
SE: Standard errors
SUA: Serum uric acid
TG: Triglycerides
TG: Triglycerides
UA: Uric acid
WC: Waist circumference
XOR: Xanthine oxidoreductase
